# Characterization of *Mycosphaerellaceae* species associated with citrus greasy spot in Panama and Spain

**DOI:** 10.1371/journal.pone.0189585

**Published:** 2017-12-13

**Authors:** Vidal Antonio Aguilera-Cogley, Mónica Berbegal, Santiago Català, Francis Collison Brentu, Josep Armengol, Antonio Vicent

**Affiliations:** 1 Laboratorio de Protección Vegetal, Centro de Investigación Agropecuaria Central, Instituto de Investigación Agropecuaria de Panamá (IDIAP), Herrera, Panamá; 2 Instituto Agroforestal Mediterráneo, Universitat Politècnica de València, Valencia, Spain; 3 Forest and Horticultural Crops Research Centre-Kade, School of Agriculture, College of Basic and Applied Sciences, University of Ghana, Legon, Accra, Ghana; 4 Centro de Protección Vegetal y Biotecnología, Instituto Valenciano de Investigaciones Agrarias (IVIA), Moncada, Valencia, Spain; Fujian Agriculture and Forestry University, CHINA

## Abstract

Greasy spot of citrus, caused by *Zasmidium citri-griseum* (= *Mycosphaerella citri*), is widely distributed in the Caribbean Basin, inducing leaf spots, premature defoliation, and yield loss. Greasy spot-like symptoms were frequently observed in humid citrus-growing regions in Panama as well as in semi-arid areas in Spain, but disease aetiology was unknown. Citrus-growing areas in Panama and Spain were surveyed and isolates of *Mycosphaerellaceae* were obtained from citrus greasy spot lesions. A selection of isolates from Panama (*n* = 22) and Spain (*n* = 16) was assembled based on their geographical origin, citrus species, and affected tissue. The isolates were characterized based on multi-locus DNA (ITS and EF-1α) sequence analyses, morphology, growth at different temperatures, and independent pathogenicity tests on the citrus species most affected in each country. Reference isolates and sequences were also included in the analysis. Isolates from Panama were identified as *Z*. *citri-griseum* complex, and others from Spain attributed to *Amycosphaerella africana*. Isolates of the *Z*. *citri-griseum* complex had a significantly higher optimal growth temperature (26.8°C) than those of *A*. *africana* (19.3°C), which corresponded well with their actual biogeographical range. The isolates of the *Z*. *citri-griseum* complex from Panama induced typical greasy spot symptoms in ‘Valencia’ sweet orange plants and the inoculated fungi were reisolated. No symptoms were observed in plants of the ‘Ortanique’ tangor inoculated with *A*. *africana*. These results demonstrate the presence of citrus greasy spot, caused by *Z*. *citri-griseum* complex, in Panama whereas *A*. *africana* was associated with greasy spot-like symptoms in Spain.

## Introduction

The ascomycete family *Mycosphaerellaceae* has a wide range of hosts and substrates. Fungi belonging to this family are characterized by producing pseudothecia, either submerged or superficially embedded in the plant tissue. The asci are typically ovoid and bitunicate, containing hyaline ascospores with a single septum. The *Mycosphaerellaceae* family includes many genera, such as *Cercospora*, *Cercosporella*, *Dothistroma*, *Lecanosticta*, *Phaeophleospora*, *Polythrincium*, *Pseudocercospora*, *Ramularia*, *Ramulispora*, *Septoria*, *Sonderhenia* and *Zasmidium*, which are under taxonomic and nomenclatural reassessment [1].

**Fig 1:**
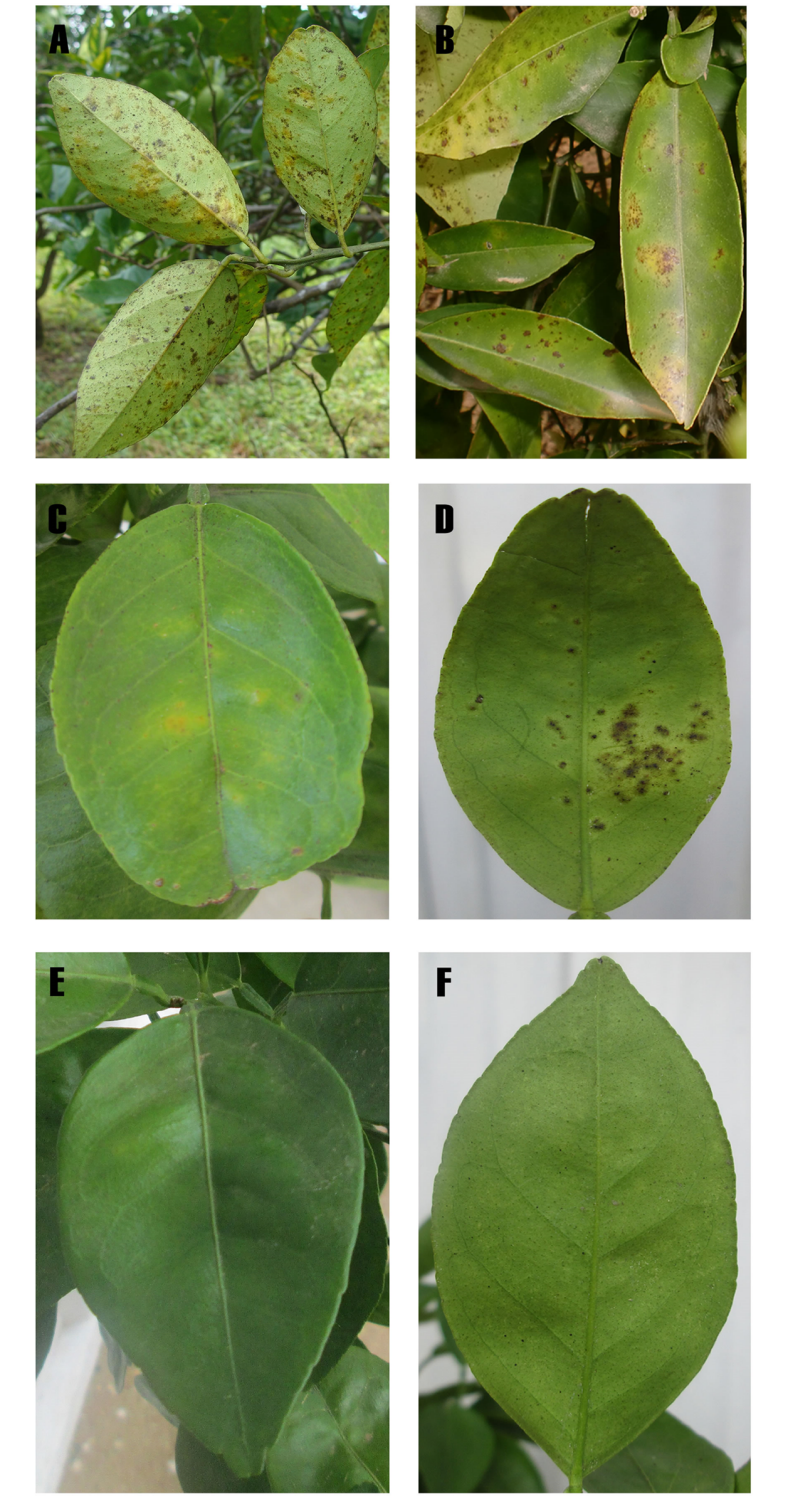
Symptoms of citrus greasy spot. A, leaves of ‘Valencia’ sweet orange in Churuquita, Panama B, leaves of ‘Ortanique’ tangor in Alzira, Spain C, chlorotic lesions on the adaxial leaf surface of ‘Valencia’ sweet orange plants inoculated with isolates of the *Zasmidium citri-griseum* complex from Panama D, necrotic pustules on the abaxial leaf surface E and F, absence of greasy spot symptoms in control plants.

Citrus greasy spot is an important fungal disease that is widely distributed in humid regions of the Caribbean Basin, although detailed aetiological studies have been conducted only in USA and Costa Rica [2]. The species *Zasmidium citri-griseum* (F.E. Fisher) U. Braun and Crous (= *Mycosphaerella citri* Whiteside) has been identified as the causal agent of greasy spot in different citrus species such as lemon (*Citrus limon*), rough lemon (*C*. *jambhiri*), grapefruit (*C*. *paradisi*), mandarin (*C*. *reticulata*), and sweet orange (*C*. *sinensis*) [3, 4]. Leaves affected by greasy spot typically show chlorosis on the adaxial surface and yellow-brown pustules on the abaxial surface (Fig 1a), resulting in premature defoliation and subsequent yield reduction. Fruit symptoms have been reported in grapefruit, consisting of minute black flecks on the peel which coalesce to form rind blotch [2].

Ascospores are the main source of inoculum of *Z*. *citri-griseum* and are produced in decomposing leaf litter following alternate wetting and drying [2]. Conidia of *Z*. *citri-griseum* have also been reported, but they are considered of minor epidemiological relevance [2, 3]. In Florida USA, peaks in ascospore production usually occur after major leaf drop, depending on rain events and temperature. Ascospores germinate and form epiphytic mycelia on the leaf surface. Subsequent penetration occurs through stomata. The disease is characterized by a relatively long incubation period, and symptoms of greasy spot are visible 4–6 months after infection [2].

Citrus greasy spot is managed mainly through foliar applications of fungicides and petroleum oils. Copper compounds have traditionally been used for greasy spot control in Florida, but triazoles and QoIs are also currently recommended. Foliar fertilizers based on iron, zinc, and manganese have also been evaluated as well as applications of urea and lime to reduce inoculum in the leaf litter [2].

Symptoms similar to citrus greasy spot have been observed in different citrus regions worldwide. In Japan, Yamada [5] reported greasy spot-like symptoms caused by *M*. *horii* Hara, which had a *Cercospora* conidial stage. In New South Wales, Australia, citrus greasy spot was indicated as caused by *Mycosphaerella* sp. [6]. In Argentina, symptoms of greasy spot have been observed on sweet orange and other *Citrus* spp., but to date the causal agent of this disease has not been described [7]. In Panama, symptoms of greasy spot are common in sweet orange, grapefruit, and Tahiti lime (*C*. *latifolia*), associated with premature defoliation and yield loss [8]. In Ghana, symptoms of greasy spot have been observed in sweet orange, grapefruit, satsuma (*C*. *unshiu*), mandarin, and rough lemon, but etiological studies were not available [9]. In China, *Z*. *fructigenum* and *Z*. *fructicola* were isolated from citrus fruits with symptoms described as greasy spot. In Indonesia, *Z*. *indonesianum* was associated with leaf spots of *Citrus* sp. [4]. Nevertheless, due to the lack of pathogenicity tests, the role of these species as citrus pathogens or endophytes is unclear.

Symptoms consisting of chlorosis and necrotic pustules on citrus leaves similar to greasy spot have also been noticed in semi-arid regions in the Mediterranean Basin, but not rind blotch symptoms on fruit. In Sicily Italy, greasy spot-like symptoms have been observed on sweet orange, lemon, grapefruit, mandarin, and their hybrids [10]. In Valencia Spain, necrotic pustules on leaves have been reported in sweet orange, mandarin, and their hybrids, with the ‘Ortanique’ tangor (*C*. *reticulata* × *C*. *sinensis*) being the most affected (Fig 1b). Both studies, in Italy and Spain, indicated that a *Mycosphaerella* sp. was associated with the disease [10, 11]. Furthermore, recent metagenomic studies in Sicily indicated that the family *Mycosphaerellaceae* was the most abundant in leaves with greasy spot symptoms [12]. Symptoms of citrus greasy spot have also been observed in sweet orange in Egypt, but in this case the species *Z*. *citri-griseum* was reported as the causal agent of the disease [13].

Considering the limited information concerning the causal agent of citrus greasy spot outside USA and Costa Rica, the objective of this work was to determine the aetiology of greasy spot symptoms in citrus-growing regions with tropical climates in Panama and semi-arid climates in Spain.

## Materials and methods

### Fungal isolates

Citrus-growing regions in Panama and Spain were surveyed between 2001 and 2013 for foliar symptoms of greasy spot. The citrus orchards sampled were visited with the owners and/or the technical crop advisers. Isolates were obtained from leaves of different citrus species showing typical symptoms of the disease, consisting of chlorosis on the adaxial surface and protuberant dark-brown pustules on the abaxial surface. Affected leaves were surface disinfested with 1% sodium hypochlorite solution for 5 min and rinsed twice in sterile distilled water. The abaxial side of the lesions was removed with a sterile scalpel, and small pieces of mesophyll were plated on potato dextrose agar (PDA) (Liofilchem, Italy) amended with 0.5 g/liter of streptomycin sulphate (Sigma-Aldrich, St. Louis, MO, USA) (PDAS), and incubated at 26–28°C in the dark for 10 to 12 days [3]. *Mycosphaerellaceae*-like colonies were transferred to malt extract agar (MEA) (Oxoid Ltd., Basingstoke, Hants, England) and monohyphal isolates were obtained by hyphal tipping. Isolations from leaf litter were carried out by attaching wetted small leaf pieces (≈5 mm^2^) with pseudothecia to the top of Petri dishes using double-sided adhesive tape (Scotch, 3M, USA) and allowing the ascospores to be ejected onto PDAS. Plates were incubated at 26–28°C in the dark for one week and examined daily. Individual germinating ascospores were selected under the stereomicroscope and transferred with a sterile needle to MEA plates, which were incubated at 25°C in the dark for two weeks [14]. All isolates were stored in 15% glycerol solution at -80°C in 1.5 ml cryovials.

A total of 38 *Mycosphaerellaceae* isolates were selected from a larger collection based on their geographical origin, citrus species, and affected tissue (Table 1). Two isolates obtained from citrus greasy spot symptoms in Ghana and two from Morocco were included for comparison. In absence of an available type culture of *Z*. *citri-griseum*, the reference isolate CBS 122455 was included in the study as well as three isolates of *Amycosphaerella africana* (Crous and M.J. Wingf.) Quaedvlieg and Crous: CBS 680.95 (holotype), CBS 110500, and CBS 110843 provided by the Westerdijk Fungal Biodiversity Institute (Utrecht, The Netherlands) (Table 1).

**Table 1:** Details of the isolates and sequences of *Mycosphaerellaceae* included in the study.

Isolate	Geographical origin				Plant species	Affected tissue	Year of isolation	Genbank accession	
	Country	Province/State	Location	Latitude/longitude				ITS	EF-1α
MC-84	Spain	Alicante	Pego	38.842650, -0.103372	*Citrus reticulata*	Canopy leaf	2008	KF963135	KY576772
MC-129	Spain	Valencia	Algemesí	39.221598, -0.438210	*C*. *sinensis*	Canopy leaf	2013	KF963139	KY576776
MC-106	Spain	Valencia	Alzira	39.142684, -0.387208	*C*. *reticulata* × *C*. *sinensis*	Canopy leaf	2011	KF963136	KY576773
MC-140	Spain	Valencia	Alzira	39.165670, -0.400005	*C*. *reticulata* × *C*. *sinensis*	Leaf litter	2013	KF963146	KY576783
MC-141	Spain	Valencia	Alzira	39.165670, -0.400005	*C*. *reticulata* × *C*. *sinensis*	Leaf litter	2013	KF963147	KY576784
MC-142	Spain	Valencia	Alzira	39.165670, -0.400005	*C*. *reticulata* × *C*. *sinensis*	Leaf litter	2013	KF963148	KY576785
MC-144	Spain	Valencia	Alzira	39.165670, -0.400005	*C*. *reticulata* × *C*. *sinensis*	Leaf litter	2013	KF963149	KY576786
MC-145	Spain	Valencia	Alzira	39.165670, -0.400005	*C*. *reticulata* × *C*. *sinensis*	Leaf litter	2013	KF963150	KY576787
MC-17	Spain	Valencia	Benimuslem	39.129963, -0.497698	*C*. *reticulata*	Canopy leaf	2005	KF963140	KY576777
MC-01	Spain	Valencia	Chiva	39.462847, -0.612966	*C*. *reticulata*	Canopy leaf	2001	KF963133	KY576770
MC-02	Spain	Valencia	Chiva	39.462847, -0.612966	*C*. *reticulata*	Leaf litter	2001	KF963143	KY576780
MC-03	Spain	Valencia	Chiva	39.462847, -0.612966	*C*. *reticulata*	Leaf litter	2001	KF963144	KY576781
MC-21^a^	Spain	Valencia	Cogullada	39.188584, -0.427264	*C*. *sinensis*	Canopy leaf	2005	KF963134	KY576771
MC-116	Spain	Valencia	L’Enova	39.066226, -0.464934	*C*. *reticulata*	Canopy leaf	2011	KF963137	KY576774
MC-136	Spain	Valencia	L’Alcúdia	39.185338, -0.520073	*C*. *unshiu*	Canopy leaf	2013	KF963142	KY576779
MC-120	Spain	Valencia	Picassent	39.371803, -0.468181	*C*. *reticulata*	Canopy leaf	2011	KF963138	KY576775
MC-102^a^	Morocco	Beni-Melal	Beni-Melal	32.356995, -6.399302	*C*. *reticulata*	Canopy leaf	2011	KF963141	KY576778
MC-105	Morocco	Beni-Melal	Beni-Melal	32.356995, -6.399302	*C*. *reticulata*	Canopy leaf	2011	KF963145	KY576782
MC-69	Ghana	Eastern Region	Kade	6.148544, -0.914819	*C*. *sinensis*	Canopy leaf	2008	KF963151	KY576746
MC-76^a^	Ghana	Eastern Region	Kade	6.148544, -0.914819	*C*. *sinensis*	Canopy leaf	2008	KF963152	KY576747
48NCCh1	Panama	Chiriqui	Potrerillo	8.667815, -82.504167	*C*. *sinensis*	Canopy leaf	2011	KF963157	KY576752
43NCCh2	Panama	Chiriqui	Rovira	8.669722, -82.511111	*C*. *sinensis*	Canopy leaf	2011	KF963153	KY576748
6NCV4	Panama	Veraguas	Bajo de la Honda	8.260184, -80.975284	*C*. *sinensis*	Canopy leaf	2010	KF963154	KY576749
9NCV4	Panama	Veraguas	El Espino	8.256232, -80.914903	*C*. *sinensis*	Canopy leaf	2010	KF963158	KY576753
4NTV1	Panama	Veraguas	Santiago	8.092347, -80.975876	*C*. *sinensis*	Canopy leaf	2010	KF963155	KY576750
15NCV4^a^	Panama	Veraguas	Alto de Piedra	8.509070, -81.101115	*C*. *sinensis*	Canopy leaf	2010	KF963161	KY576756
Myc-14	Panama	Coclé	Churuquita	8.611029, -80.266995	*C*. *sinensis*	Leaf litter	2011	KF963170	KY576765
Myc-23	Panama	Coclé	Churuquita	8.613957, -80.261073	*C*. *sinensis*	Leaf litter	2011	KF963172	KY576767
12NCC9	Panama	Coclé	Churuquita	8.611029, -80.266995	*C*. *sinensis*	Canopy leaf	2010	KF963159	KY576754
19NCC3	Panama	Coclé	El Guabal	8.617982, -80.251497	*C*. *sinensis*	Canopy leaf	2010	KF963164	KY576759
17NCC5	Panama	Coclé	Mira Flores	8.640423, -80.285906	*C*. *sinensis*	Canopy leaf	2010	KF963163	KY576758
17NCC3	Panama	Coclé	Mira Flores	8.640423, -80.285906	*C*. *sinensis*	Canopy leaf	2010	KF963162	KY576757
31TCC4	Panama	Coclé	Platanal	8.400057, -80.083242	*C*. *paradisi*	Canopy leaf	2011	KF963169	KY576764
31TCC2	Panama	Coclé	Platanal	8.400057, -80.083242	*C*. *paradisi*	Canopy leaf	2011	KF963160	KY576755
26LCC5	Panama	Coclé	Santa Clara	8.384902, -80.126427	*C*. *latifolia*	Canopy leaf	2011	KF963167	KY576762
27LCC2	Panama	Coclé	Santa Clara	8.384902, -80.126427	*C*. *latifolia*	Canopy leaf	2011	KF963168	KY576763
37LCC2	Panama	Coclé	Tambo	8.664093, -80.295722	*C*. *latifolia*	Canopy leaf	2011	KF963166	KY576761
Myc-21	Panama	Coclé	Tambo	8.664093, -80.295722	*C*. *sinensis*	Leaf litter	2011	KF963171	KY576766
38NCC2	Panama	Coclé	Tambo	8.664093, -80.295722	*C*. *sinensis*	Canopy leaf	2011	KF963156	KY576751
34NCC4	Panama	Coclé	Toabre	8.645582, -80.307244	*C*. *sinensis*	Canopy leaf	2011	KF963165	KY576760
Myc-34	Panama	Coclé	Toabre	8.645582, -80.307244	*C*. *paradisi*	Leaf litter	2011	KF963173	KY576768
Myc-36	Panama	Coclé	Toabre	8.645582, -80.307244	*C*. *paradisi*	Leaf litter	2011	KF963174	KY576769
*Zasmidium citri-griseum*									
CBS 122455	USA	Florida			*Citrus* sp.	Leaf	2003	KF901797	KF903388
CPC 15296	USA	Florida			*Citrus* sp.	Leaf	2003	KF901796	KF903385
CPC 15291	USA	Florida			*Citrus* sp.	Leaf	2003	KF901793	KF903382
CBS 116426	USA	Florida			*Musa* sp.	Leaf	2003	KF901648	KF903387
*Amycosphaerella africana*									
CBS 110843	South Africa	Western Cape	Pampoenvlei		*Eucalyptus cladocalyx*	Leaf	1994	KF901702	KF903118
CBS 680.95	South Africa	Western Cape	Stellenbosch		*E*. *viminalis*	Leaf	1994	KF901701	KF903117
CBS 110500	Australia	Western Australia	Bunbury		*E*. *globulus*	Leaf	2000	KF901516	KF903115
*Zasmidium fructicola*									
ZJUM 90	China	Fujian	Nanjing		*C*. *grandis*	Fruit	2009	KP896055	KP896102
ZJUM 89	China	Zhejiang	Linhai		*C*. *sinensis*	Fruit	2010	KP896054	KP896101
ZJUM 84	China	Zhejiang	Huangyan		*C*. *unshiu*	Fruit	2010	KP896053	KP896100
ZJUM 78	China	Hunan	Jishou		*C*. *reticulata*	Fruit	2011	KP896050	KP896097
ZJUM 58	China	Fujian	Pinghe		*C*. *grandis*	Fruit	2010	KP896047	KP896094
ZJUM 9	China	Zhejiang	Cangnan		*C*. *grandis*	Fruit	2010	KP896043	KP896090
ZJUM 68	China	Guangdong	Pingyuan		*C*. *sinensis*	Fruit	2009	KP896048	KP896095
ZJUM 80	China	Zhejiang,	Huangyan		*C*. *reticulata*	Fruit	2010	KP896052	KP896099
ZJUM 48	China	Zhejiang	Changshan		*C*. *paradisi* x *Citrus* sp.	Fruit	2010	KP896044	KP896091
ZJUM 79	China	Hunan	Jishou		*C*. *reticulata*	Fruit	2011	KP896051	KP896098
ZJUM 50	China	Zhejiang	Changshan		*C*. *paradisi* x *Citrus* sp.	Fruit	2010	KP896045	KP896092
ZJUM 77	China	Hunan	Jishou		*C*. *reticulata*	Fruit	2011	KP896049	KP896096
ZJUM 55	China	Zhejiang	Changshan		*C*. *paradisi* x *Citrus* sp.	Fruit	2010	KP896046	KP896093
*Zasmidium fructigenum*									
ZJUM 36	China	Zhejiang	Changshan		*C*. *paradisi* x *Citrus* sp.	Fruit	2009	KP896056	KP896103
ZJUM 86	China	Jiangxi	Nanfeng		*C*. *reticulata*	Fruit	2010	KP896057	KP896104
ZJUM 87	China	Jiangxi	Nanfeng		*C*. *reticulata*	Fruit	2010	KP896058	KP896105
ZJUM 88	China	Zhejiang	Linhai		*C*. *unshiu*	Fruit	2010	KP896059	KP896106
ZJUM 99	China	Zhejiang	Yuhuan		*C*. *grandis*	Fruit	2010	KP896060	KP896107
ZJUM 100	China	Zhejiang	Yuhuan		*C*. *grandis*	Fruit	2010	KP896061	KP896108
*Zasmidium indonesianum*									
CPC 15300	Indonesia				*Citrus* sp.	Leaf	2004	KF901739	KF903377
*Pseudocercospora angolensis*									
CBS 112933	Zimbabwe				*Citrus* sp.	Leaf	2000	GU269836	GU384548

^a^ Isolates not included in the phylogenetic analysis.

### DNA extraction, PCR, sequencing, and phylogenetic analyses

DNA was extracted from fungal mycelium of isolates grown on PDA at 25°C in the dark for four weeks. Colonies were scraped and mechanically disrupted by grinding them to a fine powder under liquid nitrogen using a mortar and pestle. Total genomic DNA was extracted with the E.Z.N.A. Plant Miniprep Kit (Omega Bio-Tek, Norcross, GA) and quantified using a spectrophotometer (ND-1000, NanoDrop Technologies, Wilmington, DE).

All isolates were sequenced at two loci: the nuclear ribosomal internal transcribed spacer region (ITS) and translation elongation factor 1-alpha (EF-1α). The ITS region was amplified using the primers ITS1F and ITS4. The primers EF1-688F and EF1-1251R were used to amplify part of the EF-1α. The PCR conditions were those described by Quaedvlieg et al. [14]. PCR products were separated by electrophoresis in 1.5% agarose gel (agarose D-1 Low EEO; Conda Madrid, Spain), stained with Realsafe (Real, Paterna Valencia, Spain), and visualized under UV light. Gene-ruler 100-bp DNA ladder plus was used as a molecular weight marker (Fermentas, St. Leon-Rot, Germany). The resulting products were purified and run on a DNA multicapillary sequencer (ABI Prism 3100 genetic analyser; Perkin-Elmer Applied Biosystems) by Macrogen (Amsterdam, The Netherlands).

Sequences were edited using the Sequencher software package version 5.0 (Gene Codes, Ann Arbor, MI). The loci were aligned separately using Muscle [15] and concatenated. For each locus, the most appropriate substitution model was estimated using the Akaike Information Criterion (AIC) with jModeltest2 [16]. Phylogenetic trees were estimated using Bayesian inference (critical value for the topological convergence diagnostic set to 0.01) using MrBayes version 3.2.1 [17], carried out using the substitution model selected. The Markov chain Monte Carlo method (MCMC) was initiated in the Bayesian inference from a random starting tree and run for 10^6^ generations. Posterior probabilities for each node were inferred from the resulting consensus tree. The tree was displayed with FigTree v1.4.0 [18]. For the analysis, sequences of reference isolates (Table 1) from *Z*. *citri-griseum* and *A*. *africana* were included together with other *Zasmidium* species isolated from citrus leaves in Indonesia, namely, *Z*. *indonesianum*, and from citrus fruits in China, namely, *Z*. *fructigenum* and *Z*. *fructicola* [4]. The tree was rooted to *Pseudocercospora angolensis*.

### Morphological and cultural characterization

Colony characteristics, pigment production and sporulation were evaluated on PDA, oatmeal agar (OA), spezieller nährstoffarmer agar (SNA), and MEA incubated at 25°C in the dark for 30 days. Colony colours were rated according to the charts by Rayner [19]. Since none of the isolates studied produced spores in axenic media, ascospores were collected from the same leaf litter samples where the isolates MC-140 to MC-145 from ‘Ortanique’ tangor in Alzira, Spain, and Myc-14 and Myc-23 from ‘Valencia’ sweet orange in Churuquita, Panama, were obtained (Table 1). Both orchards showed severe greasy spot symptoms. Ascospores were extracted from leaf litter as described above, but ejected onto glass slides with a drop of sterile distilled water. A total of 100 ascospores from each sample were evaluated under an optical microscope (400x) for their morphological characteristics, including length, width, colour, shape, and septation. Values of length and width of ascospores were compared with a two tailed *t*-test using the R version 3.4.0 [20].

### Effect of temperature on mycelial growth

To study the effect of temperature on mycelial growth rate, 5 mm dia. agar plugs were cut from the growing margin of 14-day-old colonies and placed in the centre of PDA plates incubated in the dark at 5, 10, 15, 20, 25, 30, and 35°C, with four replicates for each isolate (Table 1) and temperature combination. Colony diameter was measured after 21 days along two perpendicular axes, and data were expressed as radial mycelial growth rate (mm/day).

Isolates were grouped based on the results of the phylogenetic and morphological analysis. Radial mycelial growth rate data were analysed separately for each group. Several polynomial equations were fitted and compared based on the Deviance Information Criterion (DIC), which is a generalization of the AIC developed for Bayesian model comparison [21]. Parameters were estimated with Bayesian inference by Integrated Nested Laplace Approximation (INLA) with R. Default vague prior distributions were used [22]. Mean and 95% credibility interval were obtained for each parameter. Model fit was evaluated using the root mean squared error (RMSE). The optimal temperature for mycelial growth was derived from the best polynomial model obtained for each group of isolates.

### Pathogenicity tests

Due to biosecurity restrictions, only the isolates from Panama and Spain (Table 1) were included in the pathogenicity tests by performing independent experiments in each country. Isolates from Panama were inoculated in the facilities of the Instituto de Investigación Agropecuaria de Panamá (IDIAP), at Los Canelos, in Herrera Province, Panama. Inoculations with isolates from Spain were carried out at the Instituto Valenciano de Investigaciones Agrarias (IVIA) at Moncada, in the province of Valencia, Spain.

Pathogenicity tests were conducted on 2-yr-old trees using the most severely affected citrus cultivar in each country. Plants of ‘Valencia’ sweet orange grafted on ‘Swingle’ citrumelo (*C*. *paradisi* x *P*. *trifoliata*) rootstock were utilized for the isolates from Panama and plants of ‘Ortanique’ tangor grafted on ‘Carrizo’ citrange (*Poncirus trifoliata* x *C*. *sinensis*) rootstock were used for the isolates from Spain. All trees were grown individually in plastic pots (10 liter) containing potting mix (75% peat and 25% sand [vol/vol]). Potted trees were placed in a greenhouse and pruned completely to force the growth of new shoots.

Isolates were grown on PDA at 25°C in the dark for 30 days, and inoculum was prepared by flooding the agar surface with 10 ml of sterile distilled water and scraping with a spatula. The resulting mycelial suspension for each isolate was filtered through two layers of cheesecloth and adjusted to 1 x 10^4^ CFU/ml. Plants were sprayed to runoff with the mycelial suspensions using approximately 150 ml/plant, and covered with black plastic bags for 48 h. Then the bags were removed and trees were maintained at 20–35°C in the greenhouse for 24 months. In all cases, three trees were used for each isolate using a completely randomized design. Plants sprayed with sterile distilled water were used as controls. Experiments in each country were repeated once.

All leaves on each plant were observed visually after inoculation to detect symptoms of greasy spot such as chlorosis on the adaxial surface and brown pustules on the abaxial surface. Disease incidence was calculated as the proportion of symptomatic leaves, and disease severity was evaluated using the following rating scale: 0 = no lesions, 1 = 1–10 lesions/leaf, 2 = 11–20 lesions/leaf, and 3 = >20 lesions/leaf. Disease severity categories were treated as an order factor, which was modelled with a proportional odds logistic regression model using the clm function of the ordinal package for R [23]. Odds ratios based on the cumulative probabilities were calculated for each isolate. Reisolations were carried out as described above from symptomatic and asymptomatic leaves in both inoculated and control plants.

## Results

### Phylogenetic analyses

**Fig 2:**
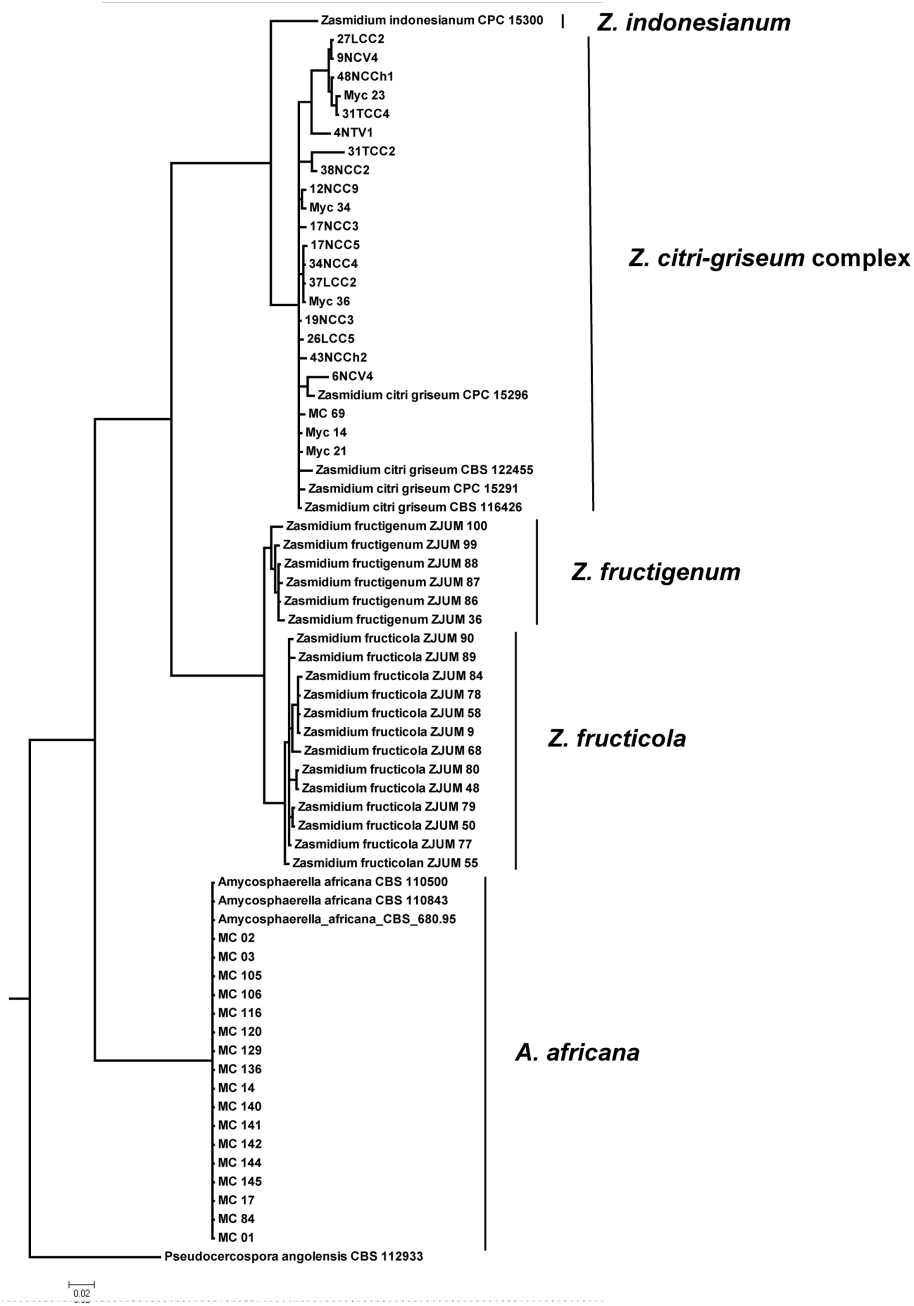
A Bayesian 50% majority rule consensus tree based on a combined ITS and EF-1α alignment, containing *Mycosphaerellaceae* isolates from citrus. Bayesian posterior probabilities support values for the respective nodes are displayed in the tree. The tree was rooted to *Pseudocercospora angolensis* (CBS112933).

Phylogenetic trees based on the combined dataset and generated by Bayesian inference separated the isolates into two groups (Fig 2). EF-1α is the locus that showed the greatest variability across the nucleotide dataset, with 29% variable sites for the entire set of data. The ITS showed a lower percentage of variable sites (13%). The isolates from Panama (*n* = 22) and Ghana (*n* = 2) were similar to *Z*. *citri-griseum* based on clustering with the sequences of the isolates CBS 122455, CBS 116426, CPC 15291, and CPC 15296 (Table 1). These isolates were found to be phylogenetically distinct over the different test loci and formed three well-supported subclusters, suggesting that this group of isolates was in fact a species complex. The isolates from Spain (*n* = 16) and Morocco (*n* = 2) were identified as *A*. *africana* based on clustering with the sequences of the reference isolates CBS 110500, CBS 110843, and CBS 680.95 (Table 1).

### Morphological and cultural characterization

**Fig 3:**
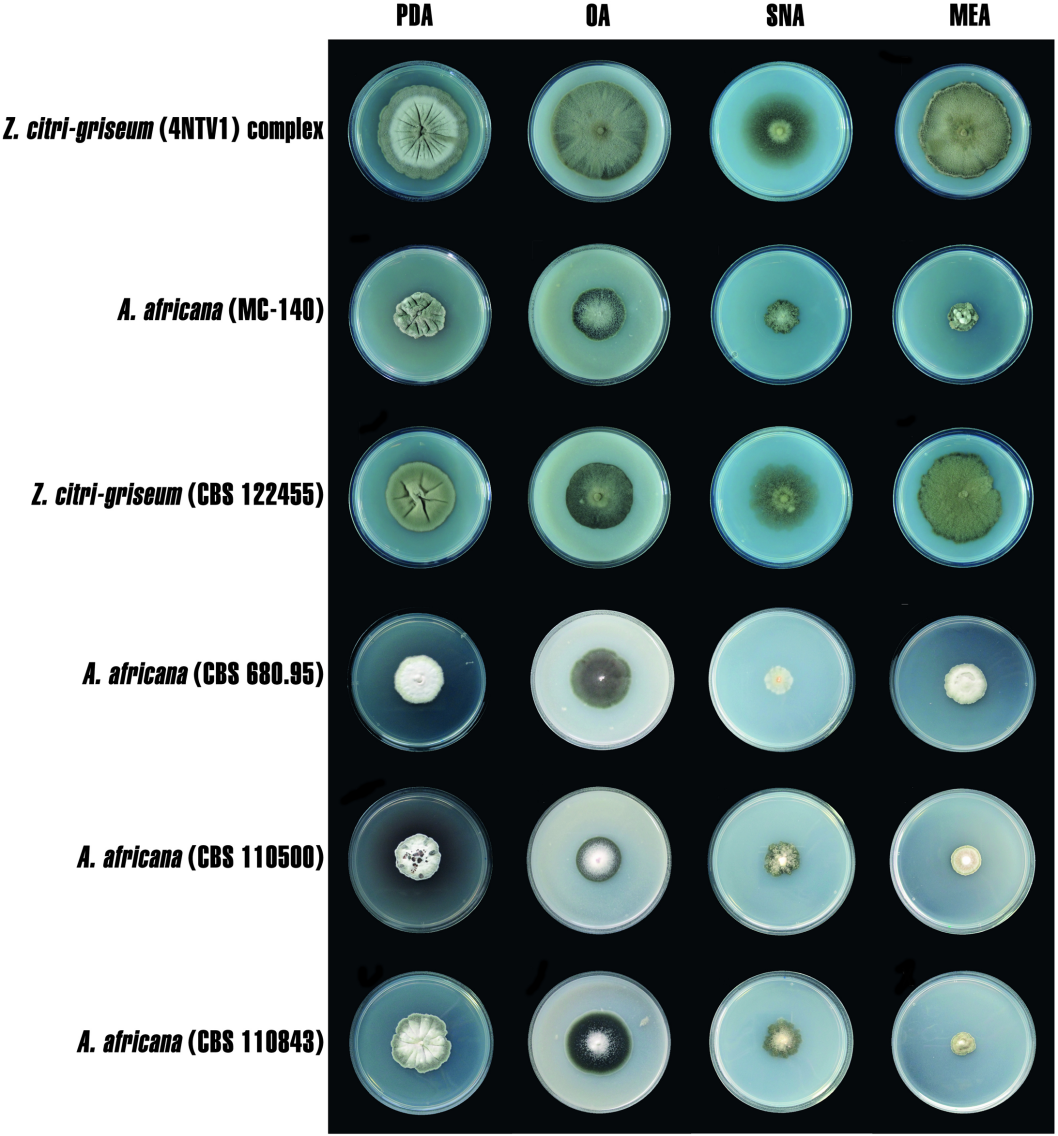
Colony morphology of *Mycosphaerellaceae* isolates: *Zasmidium citri-griseum* (4NTV1), *Amycosphaerella africana* (MC-140), reference isolate of *Z*. *citri-griseum* (CBS 122455), reference isolate of *A*. *africana* (CBS 680.95, CBS 110500, CBS 110843) on potato dextrose agar (PDA), oatmeal agar (OA), spezieller nährstoffarmer agar (SNA), and malt extract agar (MEA) incubated at 25°C in the dark for 30 days.

None of the isolates studied produced spores in axenic media. Based on the characteristics of the colonies and pigment production, the 42 isolates of *Mycosphaerellaceae* studied were divided into the same two groups defined by the phylogenetic analysis. Isolates from Panama (*n* = 22) and Ghana (*n* = 2) produced colonies on PDA with slightly lobulated margins and the upper surface varied from olivaceous-grey to mouse-grey (Fig 3). Colonies on OA showed slightly lobulated margins and the upper surface varied from grey-olivaceous to greenish-glaucous. Colonies on SNA showed slightly lobulated margins and the upper surfaces were olivaceous. Colonies on MEA also had slightly lobulated margins, but the upper surface was olivaceous-grey. The isolates 17NCC5, 34 NCC4, Myc-14, and Myc-23 produced yellow pigment, but only in PDA.

Isolates from Spain (*n* = 16) and Morocco (*n* = 2) (Fig 3) produced colonies on PDA with slightly lobulated to lobulated margins and the upper surface varied from olivaceous-grey in the margin to pale mouse-grey in the centre. Colonies on OA showed slightly lobulated margins and the upper surface varied from grey-olivaceous in the margin to greenish-glaucous in the centre. Colonies on SNA showed slightly lobulated to lobulated margins and the upper surfaces were greenish-glaucous. Colonies on MEA showed slightly lobulated to lobulated margins and the upper surface of the colonies varied from mouse-grey in the margin to pale purplish-grey in the centre. The isolates MC-02, MC-17, MC-21, MC-84, MC-105, MC-120, and MC-142 produced red pigment in PDA.

Colony characteristics of the reference isolate of *Z*. *citri-griseum* (CBS 122455) were similar to those observed in the isolates from Panama and Ghana. This reference isolate showed slightly lobulated margins in the four agar media evaluated. The colour of the upper colony surface was olivaceous-grey on PDA, grey-olivaceous on SNA, olivaceous on OA, and olivaceous-grey on MEA. Pigment formation was not observed in any of the agar media studied.

Colonies of the reference isolates of *A*. *africana* (CBS 680.95, CBS 110500, and CBS 110843) had slightly lobulated to lobulated margins on PDA, SNA, and MEA. Differences were observed in OA, with the isolates CBS 110500 and CBS 110843 having entire margins whereas the isolate CBS 680.95 had a slightly lobulated margin. Differences in colour of the upper colony surface were observed between the reference isolates of *A*. *africana* and those from Spain and Morocco. The isolate of *A*. *africana* CBS 680.95 varied from grey-lavender in the margin to rosy-buff in the centre on PDA. This same isolate was grey-olivaceous on OA, rosy-buff on SNA, and from olivaceous-buff in the margin to white in the centre on MEA. The upper colony surface of the isolate CBS 110500 of *A*. *africana* varied from umber in the margin to rosy-buff in the centre on PDA, from grey-olivaceous in the margin to pale mouse-grey in the centre on OA, was greenish-glaucous on SNA, and varied from pale mouse-grey in the margin to rosy-buff in the centre on MEA. The isolate CBS 110843 of *A*. *africana* was similar in colour to CBS 110500, but olivaceous-grey on MEA. The formation of red pigment was observed only in the isolate CBS 110500 of *A*. *africana*.

Numerous pseudothecia immersed in the abaxial surface were observed in the leaf litter samples from Panama and Spain. In both cases, pseudothecia were densely aggregated, with papillate ostioles, a diameter of up to 90 μm and with bitunicate asci. Ascospores were hyaline, slightly fusiform, with a single septum, and measured 10 to 15 x 2.5 to 5μm in the samples from Panama and 9.9 to 16.1 x 2.1 to 3.3 μm in the samples from Spain. No significant differences in ascospore length were detected (*P* = 0.724). Width of ascospores was significantly greater (*P* < 0.001) in isolates from Panama.

### Effect of temperature on mycelial growth

**Fig 4:**
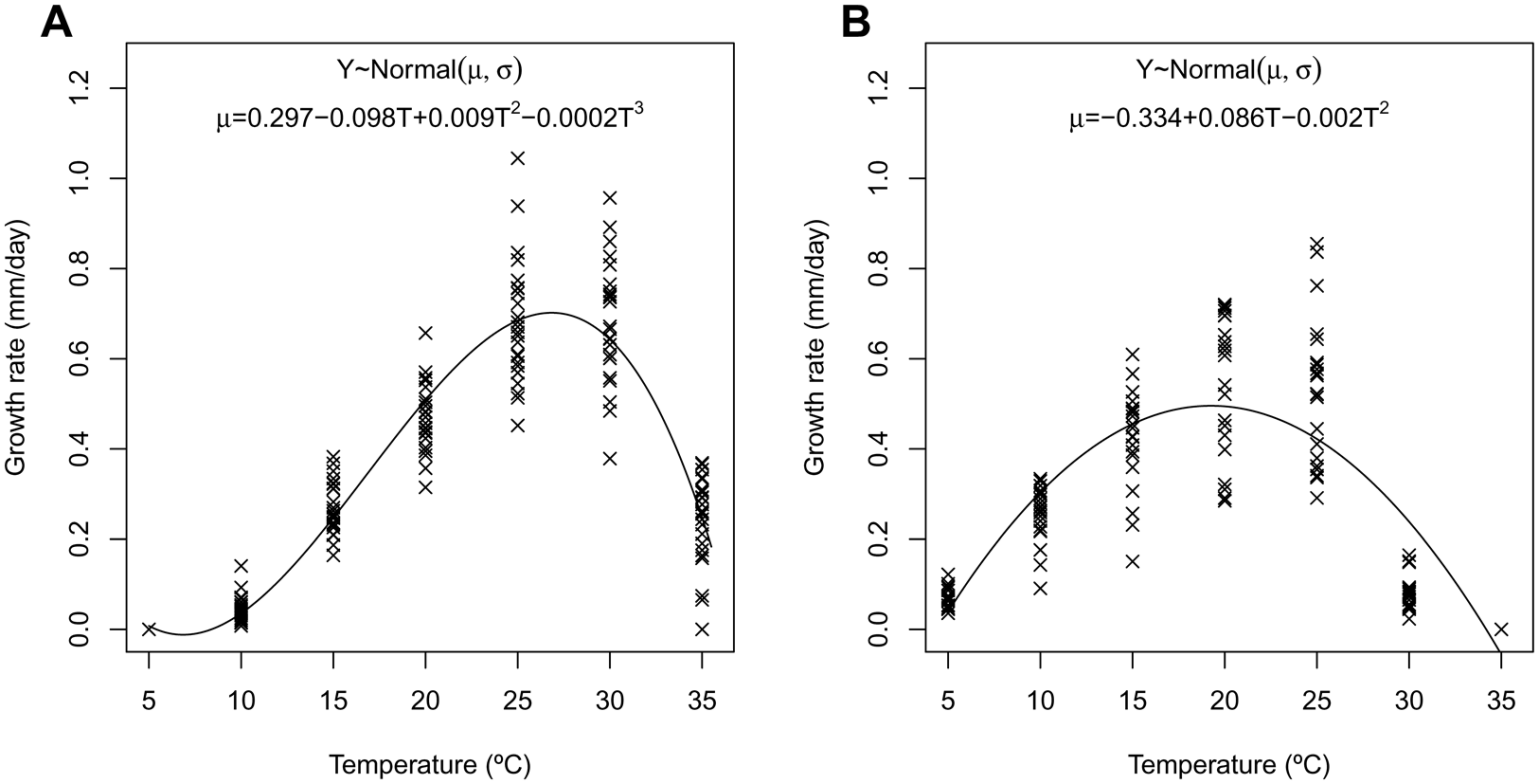
Responses to temperature (*T*) of radial mycelial growth rate (*Y*) denoted by a normal distribution of mean *μ* and standard deviation *σ*. A, *Zasmidium citri-griseum* complex isolates (*n* = 25). B, *Amycosphaerella africana* (*n* = 21).

The isolates studied showed differences in mycelial growth rate at the temperatures evaluated (Fig 4, S1 File). The best polynomial model for the isolates of *Z*. *citri-griseum* complex, including those from Panama, Ghana, and the reference isolate CBS 122455:

Y~Normal(μ,σ) (1)

$$\mu \;=\;\alpha_{0}+\alpha_{1}T+\alpha_{2}T^{2}+\alpha_{3}T^{3}$$

where *Y* = radial mycelial growth rate described by a normal distribution of mean *μ* and standard deviation *σ*, and *T* = temperature (°C). The posterior mean distribution and 95% credible intervals of the parameters were: *α*_*0*_ = 0.297 (0.191, 0.404), *α*_*1*_ = -0.098 (-0.120, -0.077), *α*_*2*_ = 0.009 (0.008, 0.010) and *α*_*3*_ = -0.0002 (-0.0002, -0.0002). The optimal temperature for mycelial growth of *Z*. *citri-griseum* complex had a mean and 95% credible interval of 26.83 (26.58, 27.09)°C, and the RMSE of the model was 0.0923.

The best polynomial model for the isolates of *A*. *africana*, including those from Spain, Morocco, and the reference isolates of *A*. *africana* (CBS 680.95, CBS 110843, and CBS 110500) was:

Y~Normal(μ,σ) (2)

$$\mu \;=\;\beta_{0}+\beta_{1}T+\beta_{2}T^{2}$$

with the same definitions of the variables as those indicated above. The posterior mean distribution and 95% credible intervals of the parameters were: *β*_*0*_ = -0.334 (-0.420, -0.250), *β*_*1*_ = 0.086 (0.076, 0.096) and *β*_*2*_ = -0.002 (-0.002, -0.002). The optimal temperature for mycelial growth of *A*. *africana* had a mean and 95% credible interval of 19.27 (18.80, 19.75°C. When comparing the two groups of isolates, those of *Z*. *citri-griseum* complex had a higher optimal temperature for mycelial growth than those of *A*. *africana*, without any overlap of their 95% credible intervals, and the RMSE of the model was 0.1265.

### Pathogenicity tests

None of the isolates of *A*. *africana* from Spain inoculated on ‘Ortanique’ tangor plants induced visible greasy spot symptoms up to 24 months after inoculation. Non-specific foliar lesions were observed on a few inoculated plants, but with very low incidence. Inoculated fungi were not reisolated from leaves of inoculated or control plants. All the isolates of the *Z*. *citri-griseum* complex from Panama induced typical greasy spot symptoms on leaves of ‘Valencia’ sweet orange 10 months after inoculation. Lesions consisted of chlorotic areas on the adaxial surface (Fig 1c) and numerous light brown to black pustules with an oily appearance on the abaxial surface (Fig 1d). Disease incidence on inoculated plants varied from 2.2% of affected leaves with the isolate 4NTV1 to 93% with the isolate 48NCCH1, both in experiment 2 (Table 2). Affected leaves within disease severity category 1 (1–10 lesions/leaf) were observed in all inoculated plants. Severity category 2 (11–20 lesions/leaf) was observed in 72% of the inoculated plants, with the isolate 6NCV4 showing the highest proportion: 35.3% in experiment 1. Leaves of severity category 3 (>20 lesions/leaf) were observed in 11 isolates, with 48NCCH1 showing the highest proportion of 21.9% of leaves.

**Table 2:** Severity of citrus greasy spot on ‘Valencia’ sweet orange plants inoculated with isolates of the *Zasmidium citri-griseum* complex from Panama and percentage of reisolation.

Isolate	Severity^a^												Reisolation (%)^c^
	Experiment 1						Experiment 2						
	0	1	2	3	Odds ratio^b^	*P*	0	1	2	3	Odds ratio^b^	*P*	
4NTV1	56	2	0	0	0.033 (0.008–0.148)	<0.0001	45	1	0	0	0.027 (0.004–0.207)	0.0005	0.0
6NCV4	14	29	30	12	12.757 (6.586–24.709)	<0.0001	53	18	7	0	0.626 (0.325–1.208)	0.1626	6.0
9NCV4	48	9	1	1	0.220 (0.098–0.496)	0.0003	13	37	17	9	7.717 (4.077–14.609)	<0.0001	40.8
12NCC9	76	30	1	0	0.374 (0.199–0.705)	0.002	38	56	7	1	1.906 (1.066–3.408)	0.030	4.1
15NCV4	53	7	0	0	0.122 (0.049–0.308)	<0.0001	56	4	0	0	0.087 (0.029–0.264)	<0.0001	0.0
17NCC3	46	8	0	0	0.161 (0.066–0.391)	<0.0001	50	19	0	0	0.449 (0.225–0.895)	0.023	0.0
17NCC5	35	61	8	0	1.733 (0.946–3.175)	0.075	18	71	9	0	3.445 (1.927–6.159)	<0.0001	51.0
19NCC3	44	14	0	0	0.291 (0.135–0.625)	0.002	32	18	5	1	0.977 (0.489–1.952)	0.947	0.0
26LCC5	55	34	4	0	0.651 (0.347–1.221)	0.181	82	14	0	0	0.206 (0.100–0.425)	<0.0001	10.2
27LCC2	54	3	0	0	0.052 (0.015–0.182)	<0.0001	81	6	0	0	0.090 (0.035–0.232)	<0.0001	0.0
31TCC2	49	6	0	0	0.114 (0.043–0.300)	<0.0001	70	4	0	0	0.070 (0.023–0.210)	<0.0001	0.0
31TCC4	18	78	8	2	3.149 (1.720–5.767)	0.0002	17	45	22	2	5.432 (2.945–10.019)	<0.0001	14.2
34NCC4	51	60	2	0	1.036 (0.573–1.875)	0.906	59	38	2	0	0.794 (0.437–1.442)	0.448	12.2
37LCC2	37	38	5	0	1.087 (0.573–2.060)	0.799	46	20	5	0	0.697 (0.359–1.352)	0.286	0.0
38NCC2	79	55	4	0	0.685 (0.383–1.224)	0.201	82	31	1	0	0.466 (0.254–0.853)	0.013	63.2
43NCCH2	53	24	0	0	0.409 (0.208–0.803)	0.009	75	6	0	0	0.097 (0.038–0.251)	<0.0001	65.0
48NCCH1	103	5	0	0	0.045 (0.016–0.125)	<0.0001	8	65	16	25	11.841 (6.590–21.279)	<0.0001	2.0
Myc-14	44	41	0	0	0.798 (0.425–1.496)	0.481	20	48	25	5	5.909 (3.245–10.762)	<0.0001	34.6
Myc-21	86	8	0	0	0.086 (0.036–0.206)	<0.0001	22	69	25	8	5.760 (3.256–10.191)	<0.0001	20.4
Myc-23	11	67	12	15	6.964 (3.726–13.015)	<0.0001	47	48	2	0	1.180 (0.656–2.124)	0.5811	51.0
Myc-36	58	26	4	0	0.496 (0.259–0.949)	0.034	26	48	13	14	4.900 (2.675–8.976)	<0.0001	85.7
Myc-34	48	12	19	2			47	22	10	1			61.2
Control	55	0	0	0			60	0	0	0			0.0

^a^ Number of leaves in each category: 0 = no lesions, 1 = 1–10 lesions/leaf, 2 = 11–20 lesions/leaf, and 3 = >20 lesions/leaf.

^b^ Odds of being above a particular severity category. Proportional odds logistic regression with the isolate Myc-34 as the reference level. In brackets 95% confidence interval.

^c^ Based on 49 leaf fragments from two experiments.

No greasy spot lesions were observed on the control plants (Fig 1e and 1f) and so all leaves were placed in the severity category 0 in both experiments, inducing serious problems of model convergence. Hence, the control treatment was not considered in the statistical analysis. The isolate Myc-34, which induced an intermediate level of disease severity in both experiments, was considered as the reference level in the proportional odds logistic regression model (Table 2). In experiment 1, when compared with isolate Myc-34, no significant differences (*P* > 0.05) in disease severity were detected with isolates 17NCC5, 26LCC5, 34NCC4, 37LCC2, 38NCC2, and Myc-14. Significantly higher (*P* < 0.05) disease severity was induced by isolates 6NCV4, 31TCC4, and Myc-23, whereas it was significantly lower (*P* < 0.05) with the other isolates evaluated. In experiment 2, when compared with isolate Myc-34, no significant differences (*P* > 0.05) in disease severity were detected with isolates 6NCV4, 19NCC3, 34NCC4, 37LCC2, and Myc-23. Significantly higher (*P* < 0.05) disease severity was induced by isolates 9NCV4, 12NCC9, 17NCC5, 31TCC4, 48NCCH1, Myc-14, Myc-21, and Myc-36, whereas it was significantly lower (*P* < 0.05) with the other isolates evaluated.

The pathogen was reisolated from 68% of the plants inoculated with isolates of the *Z*. *citri-griseum* complex from Panama, thus confirming Koch’s postulates (Table 2). The isolates 43NCCH2 and Myc-36 showed the highest percentage of reisolation with 65% and 85.7% of the leaf fragments analysed, respectively. The lowest percentage of positive reisolation was obtained with the isolates 48NCCH1 and 12NCC9 with only 2% and 4.1% of the leaf fragments analysed, respectively. Plants inoculated with the isolates 4NTV1, 15NCV4, 17NCC3, 19NCC3, 27LCC2, 31TCC2, and 37LCC2 showed typical symptoms of greasy spot, but reisolations were negative, as in the asymptomatic control plants.

## Discussion

Based on molecular, morphological, and cultural features, the isolates of *Mycosphaerellaceae* obtained from citrus leaves with greasy spot symptoms in Panama were identified as *Z*. *citri-griseum*. The phylogenetic analysis clearly separated *Z*. *citri-griseum* from *Z*. *fructigenum*, *Z*. *fructicola* and *Z*. *indonesianum*. However, the consensus tree resulted in three well-supported subclusters, suggesting that the group of *Z*. *citri-griseum* isolates was actually a species complex as previously described in this fungal family [24, 25]. In our study, the ITS was used as the primary locus together with EF-1α as a secondary identification locus, which is a strategy recommended as the most effective way of identifying many species within *Mycosphaerellaceae* and *Teratosphaeriaceae* [14]. However, an increased number of variable gene regions should be included in future research to resolve the identity of the isolates from the *Z*. *citri-griseum* complex included in this study.

The name of the causal agent of citrus greasy spot has undergone a series of changes since it was first described in Florida by Fisher [26] as *Cercospora citri-grisea* F.E. Fisher. Later, Whiteside [3] conducted a series of etiological and epidemiological studies in the same region and named the causal agent of the disease *M*. *citri*, with *Stenella* sp. for its asexual form. Sivanesan [27] described the species *Stenella citri-grisea* (F.E. Fisher) Sivan. as the asexual form of *M*. *citri*. The same names were used by Pretorius et al. [28], who considered *M*. *citri* a species complex.

Crous et al. [1] proposed the name *Z*. *citri* (Whiteside) Crous, based on the oldest anamorph name, instead of using the priority name of the teleomorph. This proposal ran against the fungal nomenclature in use at that time, but it was later accepted by the Melbourne Code, adopted in 2011. Braun and Urtiaga [29] used *Z*. *citri-grisea* (F.E. Fisher) Crous as the preferred synonym for *M*. *citri*, whereas Braun et al. [30] proposed the name *Z*. *citri-griseum* (F.E. Fisher) U. Braun and Crous, comb. nov., considering *C*. *citri-grisea* to be the oldest name, but without indicating *Z*. *citri-grisea* as a synonym. These authors questioned whether *C*. *citri-grisea* and *M*. *citri* were conspecific, because their genetic relationship has not been proven to date. Indeed, Aptroot [31] indicated that the *M*. *citri* isotype was identical to *Davidiella ammophilae*, and the topotype was in fact *M*. *punctiformis*. Nevertheless, the study by Aptroot [31] was based only on morphology and did not include molecular analyses. More recently, Huang et al. [4] described *Z*. *citri-griseum* as the causal agent of citrus greasy spot based on morphological and phylogenetic analyses, referring to *M*. *citri* as a synonym.

It has been indicated that the host range of *Z*. *citri-griseum* was limited to the *Rutaceae* [2], but this species has also been associated with necrotic leaf spots in *Musa*, *Acacia mangium*, and *Eucalyptus camaldulensis* [32]. In Thailand, *Z*. *citri-griseum* has been isolated from leaf spots in *Acacia* together with *M*. *thailandica* or *C*. *acaciae-mangii* [24]. Nevertheless, the number of isolates from non-rutaceous plants included in these studies was relatively low and so the representativeness of the surveys is difficult to assess. In our study, the ITS and EF-1α sequences of isolates from Panama were similar to those of *Z*. *citri-griseum* from *Musa* sp. and *Citrus* sp. in Florida. Nevertheless, due to the lack of pathogenicity tests in these previous studies [24, 32], the role of non-rutaceous plants as hosts or substrates of *Z*. *citri-griseum* is still unclear.

The detection of isolates belonging to the *Z*. *citri-griseum* complex in Ghana represents the first report of this pathogen in West Africa, although more extensive surveys would be needed in this country. This species was previously described as the causal agent of greasy spot in ‘Navel’ sweet orange in Egypt, based on morphology, molecular analyses, and pathogenicity tests [13]. Nevertheless, sequence data of these isolates from Egypt were not available and so it would be interesting to include them in future studies.

In the present study, isolates obtained from citrus greasy spot lesions in Spain were identified as *A*. *africana*. These isolates showed high genetic similarity with the reference isolates CBS 680.95, CBS 110500, and CBS 110843 of *A*. *africana*. This species has been previously described associated with leaf spots and defoliation in *Eucalyptus* spp. [14], although pathogenicity to this plant genus has been not demonstrated to date. Recently, a sequence type associated with an *A*. *africana* reference isolate has been found in citrus leaves with greasy spot symptoms in Sicily. Interestingly, none of the detected sequences clustered with *Z*. *citri-griseum* or allied species previously associated with citrus greasy spot in other regions [12].

The nomenclature of *A*. *africana* has also been modified during recent decades. In South Africa, Crous and Wingfield [33] reported *M*. *africana* associated with leaf spots on *E*. *deanei*, *E*. *globulus*, *E*. *grandis*, *E*. *radiata*, and *E*. *viminalis*. In Spain, Aguín et al. [34] described *M*. *africana* associated with leaf spots on *E*. *globulus*. In South Africa, Crous and Wingfield [33] described *M*. *ellipsoidea* associated with leaf spots on *E*. *cladocalyx*. The species *M*. *aurantia* has been associated with leaf spots on *E*. *globulus* in Australia and Spain [35, 36]. Recently, *M*. *africana*, *M*. *ellipsoidea*, and *M*. *aurantia* have been merged into a single species, namely *A*. *africana*, based on multigene phylogenetic analysis [14]. In our study, *A*. *africana* was isolated for the first time from citrus and, in fact, from any plant genus other than *Eucalyptus*.

Although overall morphological features allowed for a rough differentiation between the *Z*. *citri-griseum* complex and *A*. *africana*, in general the isolates studied displayed great variability. The colours of the colonies from grey to olivaceous of the *Z*. *citri-griseum* complex were similar to that of the reference isolate CBS 122455. In contrast, colony colours in *A*. *africana* did not match the reference isolates. Crous and Wingfield [33] indicated that the isolate CBS 680.95 of *A*. *africana* produced diffuse brown pigment in MEA. Likewise, Maxwell et al. [35] described the presence of brown-orange pigment in MEA with the isolate CBS 110500 of *A*. *africana*. However, in our study none of the isolates of *A*. *africana* produced pigment in MEA, including these two reference strains. The presence of yellow pigment was observed only in PDA with four isolates of the *Z*. *citri-griseum* complex from Panama, and the red pigment was seen in seven isolates of *A*. *africana* from Spain as well as in the reference isolate CBS 110500. These results illustrated the limitations of colour and pigment production as criteria for species identification in *Mycosphaerellaceae*.

The shape, size, septation, and colour of the ascospores belonging to the *Z*. *citri-griseum* complex obtained from citrus leaf litter in Panama were similar to those described by Whiteside [3]. Likewise, the morphological characteristics of *A*. *africana* ascospores obtained from leaf litter in Spain were within the range described for this species [33, 35]. Apart from ascospore width, no substantial differences were observed when comparing ascospores of the *Z*. *citri-griseum* complex from Panama and *A*. *africana* from Spain, again illustrating the limitations of morphological features to differentiate species in *Mycosphaerellaceae*.

Isolates of the *Z*. *citri-griseum* complex had an optimal growth temperature of 26.8°C, similar to the 28°C reported as the optimum for pseudothecia development in this species [2]. Isolates of *A*. *africana* showed a significantly lower optimal temperature of 19.3°C, at the lower limit of the range of the 20–25°C reported by Crous and Wingfield [33] as the optimum for this species. Different thermal adaptations were also illustrated by the fact that isolates of the *Z*. *citri-griseum* complex grew at 35°C but not at 5°C, unlike those of *A*. *africana*.

As it has been described for other fungal plant pathogens [37], the different thermal adaptations of the *Z*. *citri-griseum* complex and *A*. *africana* corresponded well with their actual geographical range. Citrus-growing regions in Panama and Ghana have tropical climates, somewhat similar to the areas in the Caribbean Basin where *Z*. *citri-griseum* has been reported. One differential characteristic of tropical climates is that the average temperature of the coldest month is seldom lower than 18°C [38]. In contrast, the climates in Spain and Morocco, where *A*. *africana* was found in our study, are semi-arid with relatively cold winters below 18°C. These biogeographical patterns suggest that isolates of the *Z*. *citri-griseum* complex are better adapted to warm citrus areas, whereas *A*. *africana* develops better in temperate citrus areas.

The results of the pathogenicity tests with mycelial suspensions clearly demonstrated that isolates of the *Z*. *citri-griseum* complex from Panama were able to induce typical greasy spot symptoms on ‘Valencia’ sweet orange plants. Disease incidence and severity obtained were consistent with previous studies using the same inoculation technique [3]. In contrast, none of the isolates of *A*. *africana* from Spain was able to induce greasy spot symptoms on plants of ‘Ortanique’ tangor, the cultivar most severely affected under field conditions. In preliminary studies, Vicent et al. [11] observed mild greasy spot-like symptoms in mandarin and grapefruit plants inoculated with mycelial suspensions of *A*. *africana*, but the fungus was not reisolated. The absence of greasy spot symptoms in our pathogenicity tests is likely to be a result of the low aggressiveness of *A*. *africana* or because inoculations with mycelial suspensions might be not adequate for this species. Nevertheless, inoculations at the optimal temperature for mycelial growth (19–20°C) should be explored. Under appropriate containment conditions, it would be interesting to conduct cross-pathogenicity tests with isolates of the *Z*. *citri-griseum* complex and *A*. *africana* from different countries and different plants, including non-rutaceous species.

## Supporting information

S1 File Values of radial mycelial growth rate at different temperatures for *Zasmidium citri-griseum* complex and *Amycosphaerella africana* isolates. (TXT)
